# Mutations in *SLC29A3*, Encoding an Equilibrative Nucleoside Transporter ENT3, Cause a Familial Histiocytosis Syndrome (Faisalabad Histiocytosis) and Familial Rosai-Dorfman Disease

**DOI:** 10.1371/journal.pgen.1000833

**Published:** 2010-02-05

**Authors:** Neil V. Morgan, Mark R. Morris, Hakan Cangul, Diane Gleeson, Anna Straatman-Iwanowska, Nicholas Davies, Stephen Keenan, Shanaz Pasha, Fatimah Rahman, Dean Gentle, Maaike P. G. Vreeswijk, Peter Devilee, Margaret A. Knowles, Serdar Ceylaner, Richard C. Trembath, Carlos Dalence, Erol Kismet, Vedat Köseoğlu, Hans-Christoph Rossbach, Paul Gissen, David Tannahill, Eamonn R. Maher

**Affiliations:** 1 Wellchild Paediatric Research Centre and Department of Medical and Molecular Genetics, University of Birmingham College of Medical and Dental Sciences, Edgbaston, Birmingham, United Kingdom; 2 Cancer Research UK Renal Molecular Oncology Group, Department of Medical and Molecular Genetics, University of Birmingham College of Medical and Dental Sciences, Edgbaston, Birmingham, United Kingdom; 3 Department of Medical Genetics, Uludag University School of Medicine, Bursa, Turkey; 4 The Wellcome Trust Sanger Institute, Cambridge, United Kingdom; 5 School of Biosciences, University of Birmingham School of Medicine, Birmingham, United Kingdom; 6 European Bioinformatics Institute, Cambridge, United Kingdom; 7 Department of Human Genetics, Center for Human and Clinical Genetics, Leiden University Medical Center, Leiden, The Netherlands; 8 Clinical Genetics and the Department of Pathology, Leiden University Medical Center, Leiden, The Netherlands; 9 Cancer Research UK Clinical Centre, Leeds Institute for Molecular Medicine, St James's University Hospital, Leeds, United Kingdom; 10 Medical Genetics Intergen Genetics Centre, Ankara, Turkey; 11 Department of Medical and Molecular Genetics, King's College London School of Medicine, Guy's Hospital, London, United Kingdom; 12 Division of Paediatric Haematology/Oncology, St. Joseph Children's Hospital, Tampa, Florida, United States of America; 13 Department of Pediatric Oncology, Gulhane Military Medical Academy, Ankara, Turkey; 14 Cranfield Health, Cranfield University, Bedford, United Kingdom; 15 West Midlands Region Genetics Service, Birmingham Women's Hospital, Edgbaston, United Kingdom; University of Washington, United States of America

## Abstract

The histiocytoses are a heterogeneous group of disorders characterised by an excessive number of histiocytes. In most cases the pathophysiology is unclear and treatment is nonspecific. Faisalabad histiocytosis (FHC) (MIM 602782) has been classed as an autosomal recessively inherited form of histiocytosis with similarities to Rosai-Dorfman disease (RDD) (also known as sinus histiocytosis with massive lymphadenopathy (SHML)). To elucidate the molecular basis of FHC, we performed autozygosity mapping studies in a large consanguineous family and identified a novel locus at chromosome 10q22.1. Mutation analysis of candidate genes within the target interval identified biallelic germline mutations in *SLC29A3* in the FHC kindred and in two families reported to have familial RDD. Analysis of *SLC29A3* expression during mouse embryogenesis revealed widespread expression by e14.5 with prominent expression in the central nervous system, eye, inner ear, and epithelial tissues including the gastrointestinal tract. *SLC29A3* encodes an intracellular equilibrative nucleoside transporter (hENT3) with affinity for adenosine. Recently germline mutations in *SLC29A3* were also described in two rare autosomal recessive disorders with overlapping phenotypes: (a) H syndrome (MIM 612391) that is characterised by cutaneous hyperpigmentation and hypertrichosis, hepatomegaly, heart anomalies, hearing loss, and hypogonadism; and (b) PHID (pigmented hypertrichosis with insulin-dependent diabetes mellitus) syndrome. Our findings suggest that a variety of clinical diagnoses (H and PHID syndromes, FHC, and familial RDD) can be included in a new diagnostic category of *SLC29A3* spectrum disorder.

## Author Summary

The histiocytoses are a group of systemic disorders usually confined to childhood and are caused by an excessive number of histiocytes which phagocytose other cells and process antigens. Although nearly a century has passed since histiocytic disorders were recognised, their pathophysiology remains largely unclear, and treatment is nonspecific. The identification of *SLC29A3* mutations as the molecular basis for a familial form of syndromic histiocytosis (FHC/RDD) confirms a direct link between Faisalabad histiocytosis and Rosai-Dorfman disease and links these disorders to other *SLC29A3*-associated phenotypes.

## Introduction

Histiocytosis encompasses a group of diverse disorders characterised by the accumulation and infiltration of monocytes, macrophages, and dendritic cells in the affected tissues. The nomenclature and classification of paediatric histiocytic disorders is complex but the most recent classification describes three major classes of histiocytoses in which the most common are class I and II [1]–[3]. Class I comprises dendritic cell disorders such as Langerhans cell histiocytosis (LCH) (also known as histiocytosis X), and includes the syndromes Letterer-Siwe disease (MIM 246400) and Hand-Schüller-Christian disease. Class II consists of macrophage-related disorders including hemophagocytic lymphohistiocytosis (HLH) which is characterised by inherited (Familial haemophagocytic lymphohistiocytosis) (FHLH) (MIM 267700) and sporadic forms of the disease. Class III comprises malignant disorders involving histiocytic lineage cells including histiocytic lymphoma and acute monocytic leukaemia. Additional, less common, histiocytic disorders included in class II are Sinus histiocytosis with massive lymphadenopathy (SHML, Rosai-Dorfman syndrome), Xanthogranuloma, Reticulohistiocytoma and a familial form previously designated as Faisalabad histiocytosis [4].

Familial forms of histiocytosis are rare [1]. FHLH can be caused by mutations in the perforin genes (*PRF1* and *PRF2*), *MUNC 13-4* and syntaxin 11 [5]. Faisalabad histiocytosis (MIM 602782) was first described as a novel autosomal recessive form of histiocytosis in a highly consanguineous family originating from Pakistan [4]. Affected individuals were born with joint deformities, sensorineural hearing loss and subsequently developed a generalized lymphadenopathy and swellings in their eyelids that were shown to contain histiocytes. The phenotype of Faisalabad histiocytosis overlaps with that of Rosai-Dorfman disease (RDD or sinus histiocytosis with massive lymphadenopathy (SHML)), a histiocytic disorder characterized by painless but protracted lymphadenopathy with lymph node histology showing large histiocytes with voluminous clear cytoplasm, rounded nuclei and emperipolesis. RDD is mostly sporadic but inherited cases can occur and Rossbach et al [6] highlighted the phenotypic similarity between familial RDD and FHC. In order to elucidate the molecular basis of FHC and familial RDD we undertook gene mapping and identification studies in three affected families.

## Results

### Mapping of a novel locus for Faisalabad Histiocytosis to chromosome 10q22.1

Three families were ascertained for gene mapping studies. Family 1 is the large consanguineous family with Faisalabad histiocytosis that was reported previously [4]. Family 2 is a Turkish family with three affected brothers with sinus histiocytosis with massive lymphadenopathy [7]. Family 3 is a Palestinian consanguineous family with 2 affected brothers and described previously [6].

A genome-wide linkage scan was performed using Affymetrix 250k SNP arrays in 8 affected individuals from the three families. The largest region of homozygosity shared by affected individuals in Family 1 was at chromosome 10q22.1 from 71,226,316 to 72,850,116 bp (rs12411657 and rs10999804). The two affected children from Family 3 also demonstrated a homozygous region at chromosome 10q22.1 between rs10509322 and rs16931177 (71,781,854–75,540,896 bp) that partially overlapped the minimal region in Family 1. Genotyping of all available family members of Families 1 and 3 with microsatellite markers D10S537 (∼72,065,347 bp) and D10S1432 (∼74,329,402 bp) confirmed linkage and provided a maximum multipoint LOD score at D10S1432 (Z_max_ = 4.79). Although the affected children in Family 2 (Turkish) were not homozygous at chromosome 10q22.1, the SNP haplotypes were identical in all 3 affected children and were therefore consistent with linkage (data not shown). A common region of autozygosity was apparent in all affected members of families 1 and 3 which gave a minimal candidate region of 1.068262 Mb between SNPs rs10509322 (71,781,854 bp) and rs10999804 (72,850,116 bp) on chromosome 10q22.1.

### Mutation analysis of candidate genes and identification of *SLC29A3* mutations

**Figure 1:**
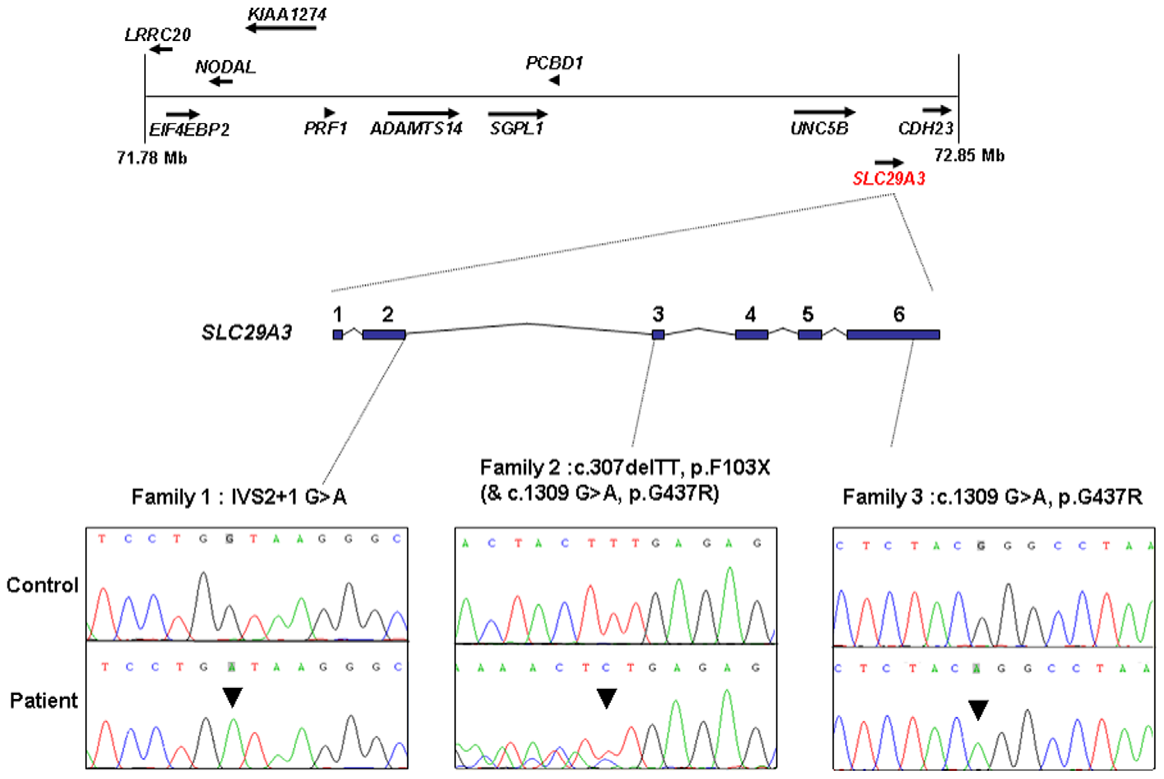
Schematic showing the minimal candidate interval on chromosome 10q22.1, positions of candidate genes taken from the Ensembl genome browser (Build 49), genomic organization of *SLC29A3*, and positions of mutations found in the 3 histiocytosis syndrome families.

**Figure 2:**
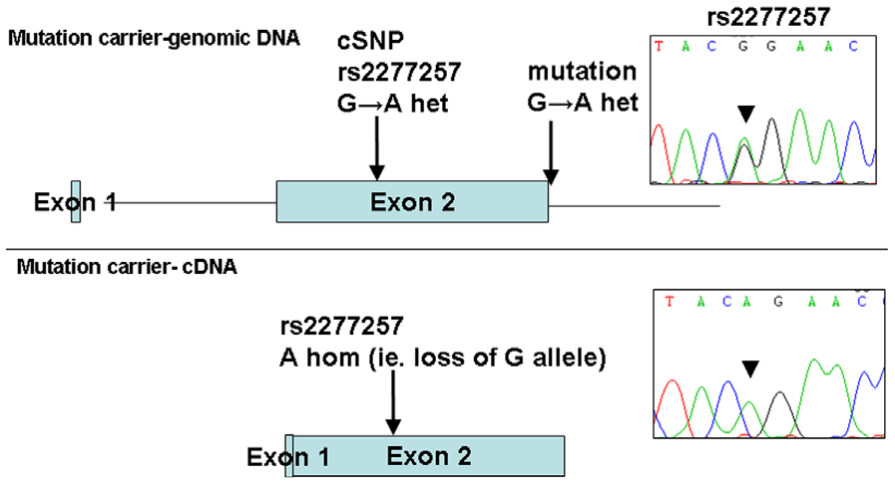
Loss of expression of mutant allele due to IVS2+1 G>A splice-site mutation identified in family 1. PCR and sequencing analysis from the genomic DNA of a mutation carrier from family 1 shows heterozygous state of SNP rs2277257 (G/A). Subsequent RT–PCR and sequencing analysis of *SLC29A3* transcript shows lack of the ‘G’ allele and therefore loss of mRNA expression.

The 1 Mb candidate region on chromosome 10q22.1 contained 11 genes (Figure 1) including Perforin (*PRF1*) which is mutated in patients with HLH (5). Direct sequencing of the coding region and flanking intronic sequences revealed no mutations in *PRF1* or in the putative tumour suppressor *UNC5B* [8]. However, direct sequencing of *SLC29A3* identified mutations in all three families (Figure 1). Thus a homozygous splice site mutation at the first base of the second intron (c.300+1G>A) was found in family 1. Lymphocyte RNA was obtained from a heterozygous carrier (VII: 9) of the c.300+1G>A mutation and RT-PCR was performed. This revealed monoallelic expression (A allele detected) of an exon 2 coding region SNP (rs2277257) although the individual was heterozygous (A/G) in genomic DNA. No abnormally sized transcripts were detected consistent with lack of expression from the mutant allele secondary to nonsense mediated RNA decay (Figure 2). A homozygous missense substitution was detected in all affected individuals in Family 3 (c.1309 G>A; p.Gly437Arg). The p.Gly437 residue occurs within the tenth transmembrane region of the protein and is highly conserved across species (data not shown). Affected individuals in Family 2 were compound heterozygotes for the p.Gly437Arg and a frameshift mutation (c.307delTT) that was predicted to produce an immediate stop codon (p.Phe103X). All mutations found in the three families segregated with the disease and were not detected in ethnically matched control chromosomes (Asian controls n = 384, Turkish controls n = 192 and Arab controls n = 84).

### Analysis of the growth-suppressing activity of *SLC29A3*

**Figure 3:**
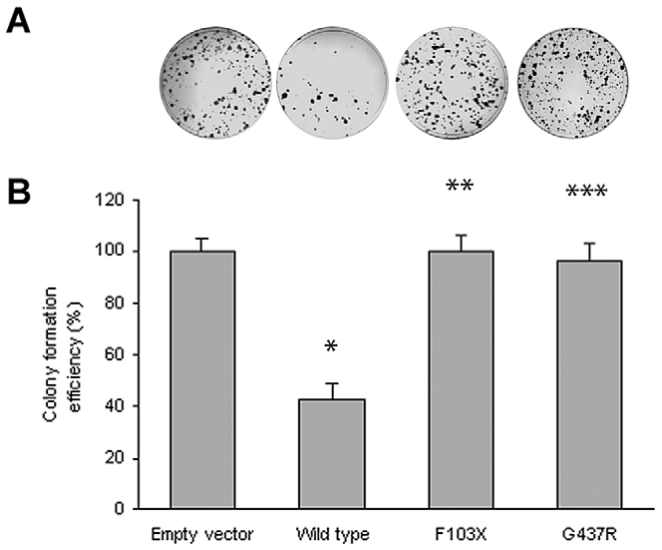
Colony formation assays of *SLC29A3*. HEK293 cells were transfected with empty vector, *SLC29A3*, *SLC29A3* ^F103X^ and *SLC29A3* ^G437R^. Each experiment was done in triplicate. The mean number of colonies counted in the empty vector plates was taken as 100%. Values are mean ± SEM from 3 controls and 3 samples. * *P* = 0.005 between wildtype *SLC29A3* vs. empty vector; ** *P* = 0.0058 and *** *P* = 0.0078 between *SLC29A3* ^F103X^ and *SLC29A3* ^G437R^ mutants resp. vs. wildtype *SLC29A3*.

Although the lymphadenopathy in Rosai-Dorfman disease is considered a reactive rather than a neoplastic process, the occurrence of two early onset cancers in the proband of Family 1 (see Table 1) raised the possibility that *SLC29A3* inactivation might cause cancer susceptibility. Hence we investigated whether *SLC29A3* might suppress cell growth using *in vitro* colony formation assays. Following transfection of a wild-type *SLC29A3* expression plasmid into HEK293 cells there was a significantly reduced number of G418-resistant colonies (mean reduction 58%, *P* = 0.005) compared with HEK293 cells transfected with an empty vector control (Figure 3). In contrast, transfection of mutant constructs (p.Gly437Arg and c.307delTT) did not significantly affect colony formation (mean change compared to empty vector 3.5% and 0% respectively).

**Table 1:** Comparison of clinical features of families with *SLC29A3* mutations from this report and those reported with H syndrome and PHID syndrome.

Clinical diagnosis	Faisalabad histiocytosis	Familial SHML	Familial Rosai Dorfman disease/Faisalabad histiocytosis	H syndrome		Pigmented hypertrichosis with insulin dependent diabetes mellitus syndrome
OMIM reference	602782			612391		
Literature reference	Moynihan et al (1998)	Kismet et al (2005)	Rossbach et al (2005)	Mohlo-Pessach et al (2008)		Cliffe et al (2009)
Inheritance	Autosomal recessive	Autosomal recessive	Autosomal recessive	Autosomal recessive		Autosomal recessive
Number of kindreds and ethnic origin	1 (Pakistani)	1 (Turkish)	1 (Palestinian)	10 (9 Arab and one Bulgarian)		5 (1 North American Caucasian, 1 Indian, 1 Pakistani and 2 Lebanese)
Skin			Hyper-pigmentation lower extremities	Hyperpigmented and hypertrichotic patches		Pigmented hypertrichotic skin lesions
Heart			Small ASD (one case)	PS, PDA		
Ear	Sensorineural deafness	Sensorineural deafness	Sensorineural deafness	Sensorineural hearing loss		No deafness
Abdomen		Hepatomegaly		Hepatosplenomegaly		Hepatosplenomegaly
Growth	Short stature	**Short stature**	Short stature	Short stature		Short stature
Endocrine	Hypogonadism			GynaeocomastiaHypogonadism		Delayed puberty
Pancreas				Occasional hyperglycaemia		IDDM in >80% of casesSevere pancreatic exocrine deficiency (two cases)
Eyes	Eyelid swellings due to histiocytic deposits	Rapidly growing orbital mass with SHML histopathology	Uveitis (1/2 cases)	Exophthalmos with normal thyroid function		
Hands	Progressive contractures of the fingers			Camptodactyly, flexion contractures of hands		No abnormality
Feet	Progressive contractures of toes			Hallux valgus with fixed flexion contractures of toe joints		
Haematological features	Bone marrow : diverse cytoplasmic inclusions in phagocytes and reticulum cells.		The bone marrow: non-clonal myeloproliferativeProcess. Numerous monocytes and histiocytes and moderate myelofibrosis.	Red cell aplasia due to myelofibrosis in one patient		
Lymph nodes	Generalised lymphadenopathy	Cervical, retropharyngeal and submandibular lymphadenopathy	Cervical, submandibular and, bilateral inguinal lymphadenopathy			Cervical, axillary and inguinal lymphadenopathy
Histopathology	Lymph node and eyelid show reactive features with small reactive lymphoid follicles and histiocytes within hyperglycaemiadilated hyperglycaemiasinuses hyperglycaemiaresembling hyperglycaemiaRosai-Dorfman disease	Lymph node: filling of lymph node sinuses with histiocytes, plasma cells and lymphocytes. Histiocytes had a benign appearance, were CD-1a negative but positive for CD68 and S-100.	Lymph node: nodal capsular fibrosis and chronic inflammation, prominent sinus histiocytes.	Skin lesions show polyclonal perivascular lymphohistiocytic infiltration with numerous plasma cells in the dermis and subcutis		
Other	Nasal mucosa swellingsContractures of the elbows and ankle**Proband: invasive ductal carcinoma grade 3 ER negative breast aged 43 years and carcinoma bladder (grade 3 papillary TCC) age 46 years**		Intra-uterine fracturesVentriculomegaly with communicating hydrocephalus and right lambdoid suture stenosis with subsequent plagiocephalyPectus carinatum			
*SLC29A3* mutations	c.300+1G>A	p.Gly437Argp.Phe103X	p.Gly437Arg	p.Gly427Serp.Gly437Argp.Leu349SerfsX56	p.Met116Arg; p.Tyr314ThrfsX91p.Gly437Arg; p.Glu444Xp.Thr449Arg	

Clinical details were taken from published reports and unpublished updated information for the three families included in the present study. Previously unreported additional information is shown in bold. Mutation nomenclature used is based on reference sequence NM_018344.4. (Abbreviations: SHML = Sinus Histiocytosis with Massive Lymphadenopathy; IDDM = Insulin dependent diabetes mellitus; ASD = atrial septal defect; PS = Pulmonary stenosis; PDA = patent ductus areteriosis; TCC = transitional cell carcinoma).

### *SLC29A3 siRNA* inhibits *SLC29A3* gene expression and promotes cell proliferation

**Figure 4:**
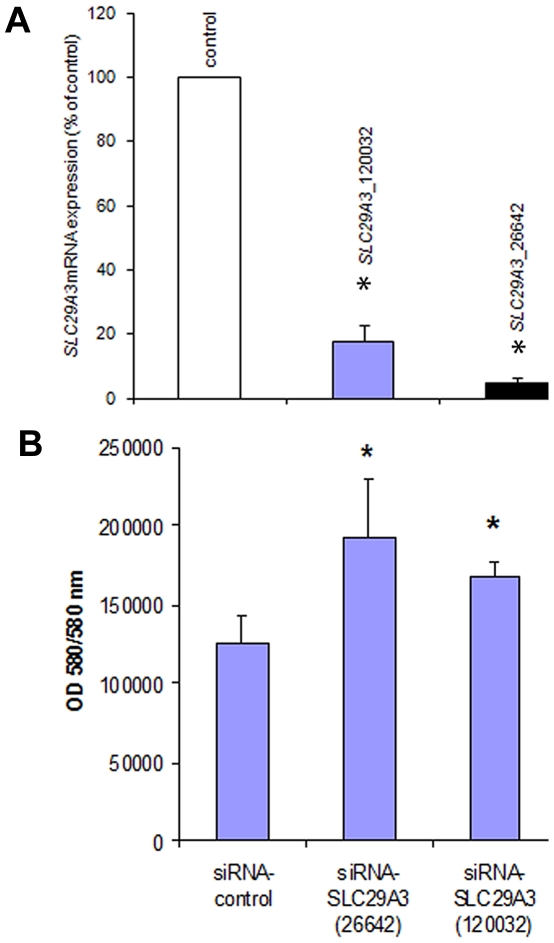
Knockdown of *SLC29A3* expression by siRNA enhanced proliferation of HeLa cells. (A) Effect of *SLC29A3* siRNA on levels of *SLC29A3* mRNA assessed by real-time quantitative PCR (qRT–PCR). HeLa cells were treated with control or *SLC29A3*- 120032 or *SLC29A3*-26642 siRNA for 72 hours. Cells were harvested and total RNA was extracted. *SLC29A3* and β-actin or *SLC29A3* and β-2-microglobulin mRNA (was examined by qRT-PCR. Values are mean ± SEM from 3 controls and 3 samples. * *P*<0.001 between HeLa cells treated with SLC29A3-siRNA versus luciferase siRNA control sequence. (B) *SLC29A3* knockdown elevates proliferation in HeLa cells. HeLa cells were transfected with siRNA designed to target *SLC29A3* (*siRNA-SLC29A3*) or control siRNA. Cell proliferation assays were performed 72 hours after siRNA transfection. Results areexpressed in fluorescence at 550 nm using 580 nm as a reference wavelength(fluorescence is directly proportional to the number of living cells). This figure represents 3 experiments.* P<0.05.

To further investigate the effect of *SLC29A3* on cell growth HeLa cells were treated with siRNA against *SLC29A3* (120032-siRNA and 26642-siRNA) or with a luciferase control sequence (control-siRNA). Compared with transfection by the luciferase siRNA sequence, *SLC29A3* mRNA levels were reduced by more than 80% in HeLa cells using 120032-siRNA and by 95% using the 26642-siRNA (Figure 4A). Knockdown of *SLC29A3* by siRNA increased cell proliferation after 72 hours when compared with control cells (control-siRNA transfection) (Figure 4B).

### *SLC29A3* expression in mouse embryos

**Figure 5:**
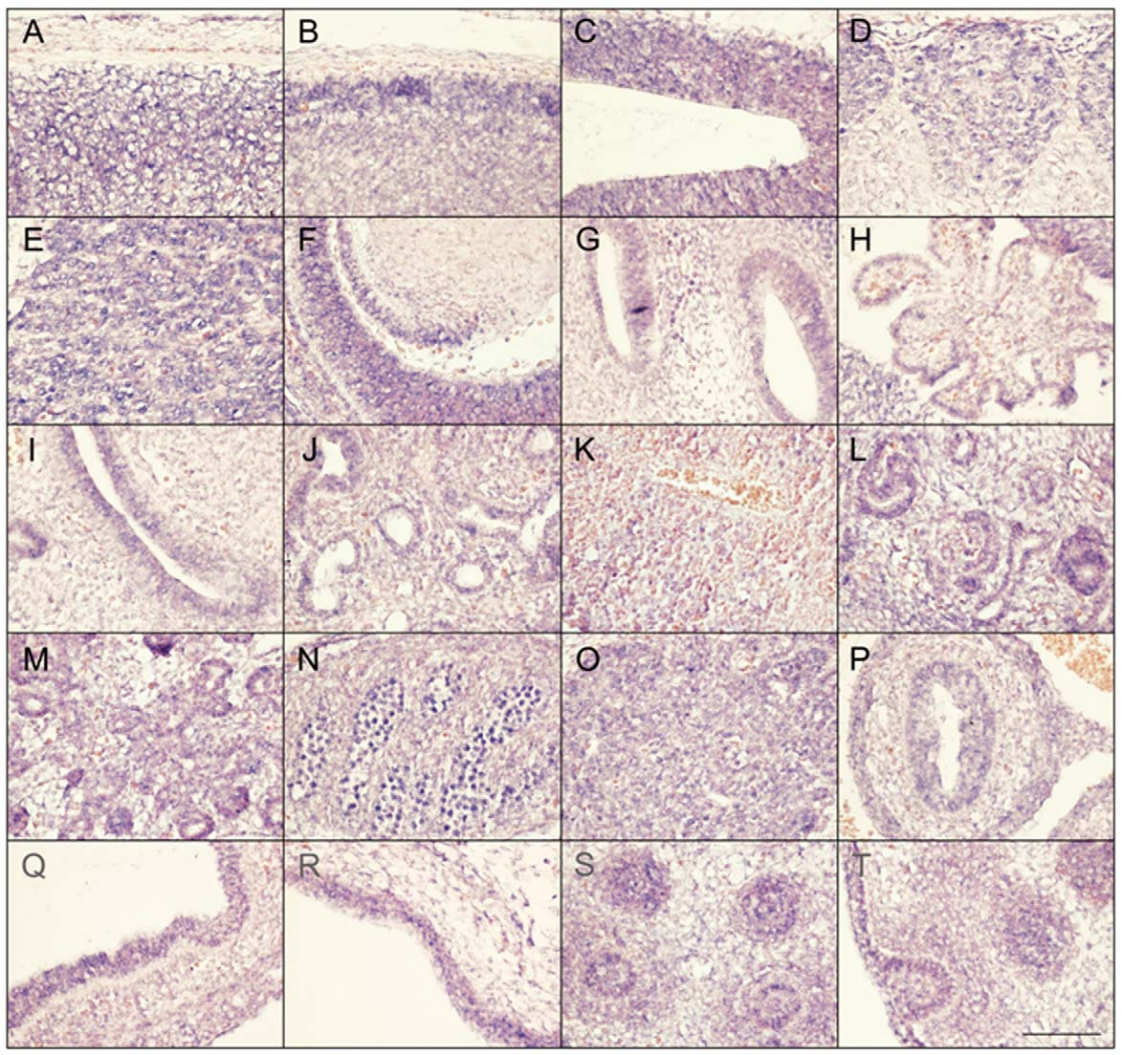
Expression of *SLC29A3* mRNA in the mouse embryo. Sagittal sections from e14.5 embryos were processed for *in situ* hybridisation followed by imaging of representative organs (hybridization signal corresponds to blue and red/pink is a general counterstain). While there is a level ubiquitous expression throughout the embryo, several areas within the central and peripheral nervous system showed increased expression levels particularly in the dorsal spinal cord (meninges and skin to the top) (A), dorsal anterior forebrain (B), dorsal posterior midbrain (C), dorsal root ganglia (D), trigeminal ganglion (E), dorsal root ganglia (E), eye and anterior lens surface (F), ear (G), choroid plexus (H), and olfactory epithelium (I). Within trunk organs localised expression is seen in the developing lung bronchioles (J), glomeruli of the kidney cortex (L), early pancreatic primordial (M), gonads (N), thymus (O), internal mucosa of the gut (P) and stomach (Q), and lower level is the liver (K). Increased *SLC29A3* expression is also seen in the outer epidermal layer of the developing posterior ventral trunk skin (R). At e14.5 trunk hair follicles are not well-developed compared, however, snout vibrissae (S,T) show elevated expression levels (a glancing more longitudinal section of an individual vibrassa can be seen in T). Scale bar in T is 100 um and applies to all images.

Faisalabad histiocytosis displays a combination of generalized and tissue specific clinical features (short stature and deafness). To reveal potential target tissues for *SLC29A3* during embryonic development, we determined the *slc29a3* expression pattern in mouse embryos by *in situ* hybridization. Within e10.5 and e12.5 embryos, weak ubiquitous *slc29a3* expression is seen throughout the embryo (data not shown). At e12.5, increased expression levels within some tissues begin to be noticeable and are more readily apparent by e14.5 (see Figure 5). Marked expression is seen throughout the central nervous system including the spinal cord and brain particularly in more dorsal most regions. A number of peripheral nervous system ganglia show elevated expression levels including the trigeminal ganglia and dorsal root ganglia. The eye shows moderate expression including the neural retina and highly localised expression at the anterior surface of the lens. In the developing ear, stronger expression can be seen in the cochlea and semi-circular canals and associated ganglia. Most striking is the expression of *slc29a3* in a variety of endodermal and epithelial-derived structures. Of particular note are the significant *slc29a3* expression levels in the luminal side of the stomach and gut and in the developing lung bronchioles, glomeruli of the cortical kidney and pancreatic primordial. The secretory surface of the choroid plexus and the olfactory epithelium also show distinct expression. Notable expression is seen in the outer layers of the developing trunk skin epidermis and within hair follicles (vibrissae) in the snout, (trunk follicles are not well developed at this time). Other sites of increased *slc29a3* expression included the thymus and developing germ cells within the testes, with lower levels in the liver.

### Mutation analysis of *SLC29A3* in breast and bladder tumours and cancer cell lines

The combination of tumour suppressor activity of hENT3 and presence of breast and bladder cancer (at ages 43 and 46 years respectively) in the proband of family 1 (VII: 7) led us to speculate that *SLC29A3* mutations might predispose to epithelial cancers and/or be mutated in sporadic breast and bladder cancer. Initially we screened for *SLC29A3* mutations in a panel of 6 breast cancer cell lines (MCF7, HCC1143, HCC1395, HCC1937, HCC1806 and HCC1419) and 37 bladder cancer cell lines. No pathogenic mutations were detected but two unclassified heterozygous variants were identified in two bladder cancer cell lines (p.Gly163Val in 5637 and p.Leu281Pro in VMCU B1). However both variants were also detected in normal controls (p.Gly163Val was detected in 3 out of 180 and p.Leu281Pro in 7 out of 174 control chromosomes tested). Screening of 48 primary breast tumour DNAs found a heterozygous germline amino acid substitution (p.Val407Met) with no loss of heterozygosity in the tumour. The variant was not detected in 400 control chromosomes and the p.Val407 residue was conserved in lower species (rat, mouse, chicken, zebrafish, *Xenopus* and *Drosophila*).

## Discussion

Although the Faisalabad histiocytosis gene was previously mapped to chromosome 11q25 in Family 1 [4], reinvestigation of the family using high-density SNP arrays excluded the original locus and identified a novel locus at chromosome 10q22.1. We then identified germline *SLC29A3* mutations in patients diagnosed with syndromic forms of histiocytosis (Faisalabad histiocytosis and familial RDD/SHML (fRDD)). RDD is classified as a reactive macrophage histiocytosis. Histopathological analysis of lymph nodes in patients with *SLC29A3*-associated FHC/RDD demonstrate prominent sinus histiocytes with large vesicular nuclei and abundant pale staining cytoplasm and emperipolesis. Langerhans cell-type histiocytes are absent, and eosinophils are rare [6]. Affected patients have a polyclonal gammopathy. Interestingly, a recent study has described germline *SLC29A3* mutations in H syndrome [9]. This autosomal recessive disorder is characterized by cutaneous hyperpigmentation, hypertrichosis, hepatosplenomegaly, heart anomalies, hearing loss, hypogonadism, short stature, hallux valgus, and fixed flexion contractures of the toe joints and the proximal interphalangeal joints [10]. Additionally, another study has recently described germline *SLC29A3* mutations in an autosomal recessive disorder, PHID (*P*igmented *H*ypertrichosis with *I*nsulin dependent *D*iabetes mellitus) syndrome, which is characterized by the childhood onset of pigmented hypertrichotic skin lesions associated with a high risk of insulin-dependent diabetes mellitus [11]. Comparison of the clinical features of families with germline *SLC29A3* mutations demonstrates overlapping features (see Table 1). Thus hyperpigmented skin lesions (histological examination demonstrates polyclonal perivascular lymphohistiocytic infiltration with numerous plasma cells in the dermis and subcutis) are common to PHID and H syndrome and were also seen in Family 3. Hearing loss and short stature is found in H syndrome and in FHC/RDD. However deafness is not a feature of PHID and diabetes mellitus and pancreatic insufficiency is restricted to PHID. Patients with H syndrome and PHID may have lymphadenopathy but do not have the life-threatening lymph node enlargement seen in FHC/fRDD [9],[11]. FHC/familial RDD, H and PHID syndromes share some common features and the phenotype of each disorder may vary between families. At present, the reason for the phenotypic variability associated with *SLC29A3* mutations is unclear. In particular the p.Gly437Arg mutation has been detected in H syndrome, PHID and FHC patients (see Table 1) suggesting that genetic and/or environmental modifiers may be implicated. We suggest that these related diseases might be viewed as manifestations of a “SLC29A3 spectrum disorder” (akin to the use of the “PTEN hamartoma tumour syndrome” to cover several clinical diagnoses caused by PTEN mutations). Further studies to determine the inter- and intrafamilial phenotypic variability of “SLC29A3 spectrum disorder” should provide insights into the molecular basis for variations in clinical phenotype and the extent to which different types of SLC29A3 spectrum disorder should be considered as clinically distinct but related disorders or as a single entity with variable expression. We found widespread expression of *slc29a3* by e14.5. It is interesting that some of the organs with prominent expression (e.g. ear, testis and skin) are affected in patients with FHC and/or H syndrome. However, we note that the clinical phenotype of *SLC29A3* spectrum disorder is more restricted than might be predicted from the expression pattern as some tissues with high expression of *slc29a3* are clinically unaffected.

Nucleoside transport in mammalian cells is performed by two types of transporters. The SLC28 family is responsible for active, sodium-dependent nucleoside transport mainly in specialised cells. In contrast the SLC29 protein family (also known as equilibrative nucleoside transporters) are passive transporters and have a wide tissue distribution. While hENT4 functions as an organic cation transporter with minimal interaction with nucleosides or nucleoside analogues [12], hENT1, hENT2 and hENT3 have been implicated in nucleoside transport and hENT3 has been reported to be a broad selectivity, low affinity nucleoside transporter that can transport adenine [13]. hENT3 was also reported to be a pH-dependent intracellular transporter with partial localization to late endosomes/lysosomes (the hENT1, 2 and 4 localise to the plasma membrane) and to contain a (DE)*XXX*L(LI) endosomal/lysosomal targeting motif [13]. However, recently it was reported that endogenous hENT3 mainly localises to the mitochondria and is predominantly a mitochondrial transporter [14]. Although germline mutations in genes encoding the mitochondrial enzymes succinate dehydrogenase and fumarate hydratase are associated with neoplasia, these disorders do not show phenotypic overlap with FHC/fRDD [15]. hENT3 has a similar broad permeant selectivity for nucleosides and nucleobases and the availability of a cytoplasmic pool of nucleosides is a key requirement for several cellular processes including the nucleoside salvage pathway and the generation of ATP/GTP for energy metabolism and signal transduction pathways [16]. In view of two early onset cancers in a proband with FHC we wondered whether *SLC29A3* might predispose to neoplasia (e.g. by altering adenosine metabolism as extracellular adenosine has been reported to induce apoptosis and proliferation in gastric, leukaemia and hepatic cancer cell lines [17]–[20]). We found that ectopic expression of *SLC29A3* suppressed cell growth *in vitro* but did not find conclusive evidence of *SLC29A3* inactivation in breast or bladder cancers. Further clinical studies are required to determine whether the frequency of neoplasia in SLC29A3 spectrum disorder is increased or whether the findings in the FHC family are coincidental. Interestingly, knockdown of the *Drosophila* orthologue of *SLC29A3* (dENT1) results in a variety of phenotypes ranging from lethality in pupal and pharate adult stages (with complete knockdown) or adult flies with abnormal sensory bristle development (mild knockdown). The latter phenotype has been linked to abnormalities of insulin signalling pathway, and, in contrast to the growth suppressive effects of hENT3 in human cell line, dENT1 appeared to be a positive promoter of cell size or number. The identification of genes for rare familial syndromes can provide insights into the molecular pathogenesis of more common disorders. Hence the analysis of the *SLC29A3* pathway in sporadic Rosai-Dorfman disease, will be of interest. It is unclear if SLC29A3 might prove to be relevant to the pathogenesis of other non-familial histiocytic disorders but we note that the use of nucleoside analogues for the therapy of Langerhans cell histiocytosis has been suggested previously [21].

## Methods

### Ethics statement

This study was conducted according to the principles expressed in the Declaration of Helsinki. The study was approved by the South Birmingham Research Ethics Committee (equivalent to the Institutional Review Board of Birmingham Women's Hospital) reference number CA/5175. All patients provided written informed consent for the collection of samples and subsequent analysis.

### Patient ascertainment

Three families previously published were ascertained for gene mapping studies [4],[6],[7].

### Gene mapping

A genome-wide linkage scan was carried out using the Affymetrix 250K SNP chip in individuals affected with Faisalabad Histiocytosis (Family 1; VI:6, VII:3 and VII:7); (Family 2; all 3 affected individuals); (Family 3; 2 affected individuals). This scan excluded linkage to chromosome 11q25 and identified a single region of extended homozygosity at chromosome 10q22.1 shared by all three affected individuals of family 1 and the two affected individuals of family 3. In addition all affected individuals of family 2 shared a disease haplotype for this region at chromosome 10. These homozygous regions were further analysed by typing microsatellite markers (D10S537 and D10S1432) in all family members from whom DNA was available.

### Mutation analysis

We identified positional candidate genes using data from the National Centre for Biotechnology Information (NCBI) and University of California Santa Cruz human genome databases. Sequencing was performed using standard methods on an ABI 3730 automated sequencer. For *SLC29A3*, we designed primer pairs using exon-primer to amplify exons 1–6, exon 6 was amplified in 2 fragments. PCR primer sequences are available on request.

Genomic DNA from 6 breast cancer cell lines (MCF7, HCC1143, HCC1395, HCC1937, HCC1806 and HCC1419), 48 primary breast tumours, 37 bladder cancer cell lines, were used for *SLC29A3* mutation screening.

### RT–PCR

Total RNA was extracted from human lymphocytes using the RNeasy Mini Kit (Qiagen). cDNA was synthesized using random primers and AMV reverse transcriptase using the Promega reverse transcription system A3500 according to manufacturers' instructions (Promega). RT-PCR was performed using the following primers: SLC29A3_1_FOR (5′- AGCCCAGTGGTCCTGGC-3′) and SLC29A3_REV (5′-CTAGATGAGGTGCACCAGGAGGGT-3′), followed by a nested primer set SLC29A3_INT_FOR (5′-ATGGCCGTTGTCTCAGAGG-3′) and SLC29A3_INT_REV (5′-GTAGAGGAGGGCCAG-3′).

### Plasmid constructs

A wild type construct of the full coding region of human *SLC29A3* was PCR amplified from the IMAGE clone 40116514 with primers: 5′- GCTAGTCGACTTATGGCCGTTGTCTCAGAGG-3′ and 5′- CGATGGTACCCTAGATGAGGTGCACCAGGAGGGT-3′. Forward primers contained a *Sal*I restriction site and reverse primers contained a *Kpn*I site to allow subcloning of the PCR fragments into the pEGFP-C2 vector (BD Biosciences Clontech). The mutations p.Gly437Arg and c.307delTT were created using the QuikChange site-directed mutagenesis kit (Stratagene) with primers: SLC29A3_G437R_FOR (5′-CTGGCCCTCCTCTACAGGCCTAAGATTGTGC-3′), SLC29A3_G437R_REV (5′-GCACAATCTTAGGCCTGTAGAGGAGGGCCAG-3′), SLC29A3_307delTT_FOR (5′-GACATCCTGAACTACTGAGAGCTACCTTGC-3′) and SLC29A3_307delTT_REV (5′-GCAAGGTAGCTCTCAGTAGTTCAGGATGTC-3′). All plasmid constructs were verified by sequencing.

### Cell culture

HeLa and HEK293 cells were routinely maintained in DMEM (Sigma) supplemented with 10% fetal bovine serum at 37°C, 5% CO_2_.

### Colony formation assay

10 µg of empty vector or plasmid construct was transfected into 5×10^5^ HEK293 cells using Lipofectamine 2000 (Invitrogen) according to manufacturers instructions and 48 h later cells were seeded in a serial dilution and maintained in DMEM and 10% fetal bovine serum supplemented with 1 mg/ml G418 (Invitrogen). Surviving colonies were stained with 0.4% crystal violet (Sigma) in 50% methanol, 14 to 21 days after initial seeding, and counted. Each transfection was carried out in triplicate.

### *SLC29A3* siRNA

HeLa cells were plated in 6-well plates at a density of 2×10^5^ cells per well prior to transfection with siRNA oligonucleotides. After 24 h cells were transfected with two different predesigned siRNAs (SLC29A3-120032 targeted to exon 6 and SLC29A3-26642 targeted to exon 2 of *SLC29A3*) (Ambion) at a final concentration of 3mM using INTERFERin (Polyplus transfection) and OPTIMEM (Invitrogen). The firefly Luciferase (GL2) (CUG ACG CGG AAU ACU UCG A) was used as a siRNA control. Transfected cells were collected after 3 days incubation and analysed for mRNA expression.

### Real-time quantitative RT-PCR (RQ–PCR)

To determine whether *SLC29A3* mRNA expression was altered in siRNA transfected HeLa cells a RQ-PCR assay was developed using TaqMan technology and an ABI 7900 HT analyser (Applied Biosystems, Warrington, UK). RNA samples were isolated and reverse-transcribed (as described above) and amplified with the relevant primers using SYBR-Green based technology (SensiMix, Quantace). Control samples were serially diluted to encompass the range of expression levels encountered in HeLa cells. Gene expression was quantified in terms of gene normalized copy number (NCN) which was derived from the relevant standard curve. The cDNA was diluted 1 in 5 for all samples and controls. A control sample was then further diluted to give 1 in 5, 1 in 10 and 1 in 20 dilutions to make a standard curve. PCR reactions were set up with primers for *SLC29A3* (SLC29A3_FOR_5′-ATGGCCGTTGTCTCAGAGG-3′ and SLC29A3_REV 5′-GGCAACGGCAAGGTAGCTCTC-3′), and *β-Actin* (β-Actin_FOR 5′-GCGGGAAATCGTGCGTGACATT-3′ and β-Actin_REV 5′-GATGGAGTTGAAGGTAGTTTCGTG-3′) and *β*-2-microglobulin (β-2M_FOR 5′-CCTTGAGGCTATCCAGCGT-3′ and β-2M_REV 5′-CCTGCTCAGATACATCAAACATG-3′) which acted as housekeeping genes. A 25µl real-time PCR reaction mixture was then set up using SensiMix (2×), 2.5 µl cDNA and 0.2 µM gene-specific primers for *SLC29A3*, *β-Actin and β-2-microglobulin*. Following the PCR the specificity was examined on a 4% agarose gel. The real-time PCR result was then analysed using the SDS 2.3 software and checked for a bimodal dissociation curve or abnormal amplification plot. Each reaction was set up in triplicate including a non-template control.

### Cell proliferation assay

Reverse transfection of siRNA duplexes was performed at 3 nM per well in a 96-well plate. Briefly 5×10^3^ HeLa cells (in 125 µl media) were plated in 96-well plates and transfected with INTERFERin and siRNA oligonucleotides designed to target *SLC29A3* (120032-siRNA and 26642-siRNA) or control luciferase sequence as described previously. Then cell viability was assessed after 48 hours using CellTiter-Blue cell viability assay (Promega) according to the manufacturer's instructions.

### Analysis of *slc29a3* expression in mouse embryogenesis

*In situ* hybridization was carried out as described previously [22] using an automated Ventana Discovery platform (Tuscon, Arizona) with minor modifications. C57BL/6J-Tyr^C-Brd^ albino mouse embryos were collected humanely under UK animal licensing laws by a schedule 1 procedure. Embryos were formalin-fixed and paraffin-embedded followed by sectioning at 8mm in the sagittal orientation. For comprehensive anatomical coverage of all embryonic organs at e14.5 (EMAP ontology terms see http://www.emouseatlas.org/Atlas/intro.html), every 10^th^ section was processed for *in situ* hybridisation. Following hybridization, labelled antisense RNA probe for the mouse *slc29a3* gene (from position 1912 to 2230, RefSeq NM_023596) was detected on the instrument using sheep anti-digoxygenin alkaline phosphatase Fab fragments (Roche) followed by incubation with BCIP/NBT (Roche). Sections were counterstained with neutral red, mounted in Eukitt (SigmaAldrich) and photographed at 40× on a Zeiss Axiskop 2 compound microscope. These *in situ* hybridisation experiments formed part of a high-throughput studt at the Wellcome Trust Sanger Institute in which over 400 genes on ∼68,000 sections were studied (see www.embryoexpress.org) in which positive and negative *in situ* hybridization controls were routinely performed.
